# Sampling and Analytical Method for Alpha-Dicarbonyl Flavoring Compounds via Derivatization with* o*-Phenylenediamine and Analysis Using GC-NPD

**DOI:** 10.1155/2016/9059678

**Published:** 2016-05-10

**Authors:** Stephanie M. Pendergrass, Jeffrey A. Cooper

**Affiliations:** ^1^National Institute for Occupational Safety and Health Centers for Disease Control and Prevention, Cincinnati, OH 45226, USA; ^2^Bureau Veritas North America, Novi, MI 48375, USA

## Abstract

A novel methodology is described for the sampling and analysis of diacetyl, 2,3-pentanedione, 2,3-hexanedione, and 2,3-heptanedione. These analytes were collected on* o*-phenylenediamine-treated silica gel tubes and quantitatively recovered as the corresponding quinoxaline derivatives. After derivatization, the sorbent was desorbed in 3 mL of ethanol solvent and analyzed using gas chromatography/nitrogen-phosphorous detection (GC/NPD). The limits of detection (LOD) achieved for each analyte were determined to be in the range of 5–10 nanograms/sample. Evaluation of the on-tube derivatization procedure indicated that it is unaffected by humidities ranging from 20% to 80% and that the derivatization procedure was quantitative for analyte concentrations ranging from 0.1 *μ*g to approximately 500 *μ*g per sample. Storage stability studies indicated that the derivatives were stable for 30 days when stored at both ambient and refrigerated temperatures. Additional studies showed that the quinoxaline derivatives were quantitatively recovered when sampling up to a total volume of 72 L at a sampling rate of 50 cc/min. This method will be important to evaluate and monitor worker exposures in the food and flavoring industry. Samples can be collected over an 8-hour shift with up to 288 L total volume collected regardless of time, sampling rate, and/or the effects of humidity.

## 1. Introduction

In August 2000, the National Institute for Occupational Safety and Health (NIOSH) received a request for technical assistance (HETA # 00-0401) in an investigation of severe obstructive lung disease (*bronchiolitis obliterans*) in former workers of a microwave popcorn plant in Missouri [1]. NIOSH was asked to investigate a cluster of past and present employees experiencing severe respiratory symptoms after working in microwave popcorn processing facilities over a period of 3 months to 3 years [2]. A NIOSH medical and environmental survey at the plant in November 2000 demonstrated a strong exposure-response relationship between quantities of estimated cumulative exposure to* diacetyl* (a volatile butter flavoring chemical contaminating the air in the plant) and the frequency of airway obstruction on spirometry tests [1].

NIOSH method 2557, an air sampling method that uses Anasorb Carbon Molecular Sieve (CMS) sorbent tubes, was developed based on an urgent need for a method to collect and quantitate exposures and evaluate subsequent engineering control effectiveness [3]. This method was used extensively in the field for a number of years. Subsequent field evaluation work suggested a tendency of NIOSH method 2557 to underestimate the true concentration of diacetyl in air [4]. Additional laboratory studies identified that this method had reduced recoveries when samples were collected in moderate-to-high humidity environments. A NIOSH laboratory-based study and a chamber study with generated atmospheres established a correction method for previously collected data with the initial NIOSH method [5]. Concurrently, the Occupational Safety and Health Administration (OSHA) developed method PV2118 that collected diacetyl on a silica gel sorbent. While the method exhibited good storage stability for diacetyl, it had limitations in sampling time/volume because of the collection of water during air sampling [6].

In an effort to address the humidity concerns encountered by the NIOSH and OSHA methods, OSHA developed another method for the collection and analysis of diacetyl on specially dried silica gel tubes (2 tubes in series) [7]. By using the dried silica gel tubes in series, this OSHA method addressed migration issues encountered when a single silica gel tube was used. All of these methods utilize gas chromatography equipped with flame ionization detection (GC/FID) for sample analyses.

In 2011, NIOSH published a draft criteria document titled “Criteria for a Recommended Standard: Exposure to Diacetyl and 2,3-Pentanedione” that contained a draft NIOSH Recommended Exposure Limit (REL) of 5 pbb, 8 hr-TWA for diacetyl [8]. The criteria document recommended OSHA method 1012 for sampling diacetyl exposures. This method utilizes* o*-(2,3,4,5,6-pentafluorobenzyl) hydroxylamine hydrochloride (PFBHA) to derivatize diacetyl followed by analysis using gas chromatography with electron capture detection (GC-ECD) [9]. OSHA method 1012 has limitations in sampling time and capacity due to the potential collection of water during air sampling, as well as an extended derivatization time up to 36 hours.

To address the limitations in diacetyl sampling, a research protocol was designed based upon the derivatization of diacetyl (which was subsequently applied to analogous alpha-dicarbonyl compounds) with* o*-phenylenediamine (*o*-PDA). Several research groups have documented the conversion of alpha-dicarbonyl compounds into the corresponding quinoxalines using* o*-PDA [10–12].

Therefore, the focus of this research project was to develop a method for the collection, derivatization, and stabilization of diacetyl and the other alpha-dicarbonyl compounds (2,3-pentanedione, 2,3-hexanedione, and 2,3-heptanedione) as quinoxaline derivatives.

## 2. Methods

### 2.1. Apparatus

Gas chromatographic (GC) analyses were conducted using a Hewlett Packard Model 5890 Series II GC with a nitrogen/phosphorus detector (NPD) (Agilent Tech., Avondale, PA) equipped with a 30 m RTX-5 fused silica capillary column (0.25 mm ID, 1 *μ*m film) (Restek Corp., Bellefonte, PA).

Baseline separation and optimal resolution of diacetyl, 2,3-pentanedione, 2,3-hexanedione, and 2,3-heptanedione from the excess derivatizing reagent were achieved using the following parameters. The GC oven temperature program was ramped up from 50°C (held 1 min) to 200°C (10°C/min) and held for 2 min. The injection port temperature was set at 240°C, the detector temperature at 300°C, and the carrier gas (helium) to a flow rate of 1.36 mL/min. The injection solvent was ethanol, which was also used as the method desorption solvent. A splitless GC injection port liner was used and 1 *μ*L aliquot was injected.

### 2.2. Reagents

Diacetyl (97%, CAS # 431-03-8),* o*-PDA (99.5%, CAS # 95-54-5), 2,3-dimethylquinoxaline (97%, CAS # 2379-55-7), 2,3-pentanedione (97%, CAS # 600-14-6), 2,3-hexanedione (≥93%, CAS # 3848-24-6), 2,3-heptanedione (≥97%, CAS # 96-04-8), and ethanol (99.5%, CAS # 64-17-5) were purchased from Sigma-Aldrich Chemical Co. (Milwaukee, WI).

Commercially available silica gel sorbent tubes (SKC # 226-183) and specially prepared* o*-PDA-treated silica gel sorbent tubes (SKC # CPM021109-001) were obtained from SKC, Inc. (Eighty Four, PA). The commercially available silica gel sorbent tubes contain two sections of silica gel (600 mg front section and 600 mg back section). The* o*-PDA- (nominally 0.1% by weight) treated silica gel sorbent tubes contain two sections of treated silica gel (520 mg front section, a PUF separator, and 260 mg back section). Ethanol (99.5%, CAS # 64-17-5) was used as the solvent for all spiking solutions and as the eluting solvent. For all sorbent tubes, the front section (A) and back section (B) were desorbed separately in 3 mL of ethanol in autosampler vials (sealed) and placed on a shaker for 90 minutes to facilitate desorption. Analyte spikes, depending on the study, were placed on the front section of the sorbent tube, or onto the initial glass wool plug, or from generated aerosols. For each concentration level evaluated, six samples (*N* = 6) were prepared. A Teflon magnetic stir bar (12.7 mm × 7.9 mm, VWR, Inc.) was placed in each vial. After the desorption period, a portion (1 mL) of each sample was transferred to 2 mL autosampler vials for analysis using GC-NPD (1 *μ*L injection).

### 2.3. Procedures

In order to address the identified limitations of current methods, a number of laboratory evaluations were conducted: (a) determination of LOD and Limit of Quantitation (LOQ), (b) determination of the efficacy of the postsampling derivatization of diacetyl collected on large untreated silica gel tubes, (c) determination of diacetyl, 2,3-pentanedione, and 2,3-hexanedione recovery from* o*-PDA-coated silica gel sorbent, (d) determination of the effects of high humidity on the derivatization process, (e) determination of the maximum collection capacity of the coated silica gel sorbent, and (f) determination of analyte storage stability.

#### 2.3.1. LOD/LOQ Determination

Using GC-NPD, eight standards (analyzed in duplicate) were prepared and derivatized on-tube ranging from 2.65 ng/mL to 662.5 ng/mL for diacetyl, from 10 ng/mL to 100.7 ng/mL for 2,3-pentanedione, and from 5 ng/mL to 201.6 ng/mL for 2,3-hexanedione.

For LOD and LOQ determination, analytical standards were prepared by serial dilution for diacetyl, 2-pentanedione, and 2,3-hexanedione solutions and 1 *μ*L aliquots were spiked directly onto the sorbent tube. After equilibration, the sorbent sections were desorbed in 2 mL of ethanol for 60 minutes [13].

#### 2.3.2. Recovery Study (Untreated Silica Gel Sorbent Tubes)

Initially, untreated silica gel sorbent tubes were prepared for desorption efficiency (DE) studies after the determination of the method LOD/LOQ using GC-NPD. Spikes were prepared at the following levels: 0.0955 *μ*g, 9.55 *μ*g, 95.5 *μ*g, 239 *μ*g, and 478 *μ*g.

#### 2.3.3. Recovery Study (*o*-PDA-Treated Silica Gel Sorbent Tubes)

For the initial desorption efficiency study, where the custom-made and* o*-PDA-treated silica gel tubes (SKC # CPM021109-001) containing 0.1% * o*-PDA by weight were used, the desorption efficiencies for diacetyl, 2,3-pentanedione, 2,3-hexanedione, and 2,3-heptanedione were evaluated. Spikes were prepared ranging from approximately 0.1 *μ*g to 500 *μ*g (10 to 100 *μ*g for 2,3-heptanedione) and are listed in Table 1. The ensuing sample preparation and analyses were the same as described in the previous section.

#### 2.3.4. Low-Level Recovery Studies

To further define the lower sample recovery limits, a low-level recovery study (0.1 to 1 *μ*g) was conducted for each analyte. Using the custom-made, unwashed, and dried* o*-PDA-treated silica gel tubes (SKC # CPM021109-001), diacetyl, 2,3-pentanedione, 2,3-hexanedione, and 2,3-heptanedione were evaluated.

#### 2.3.5. Studies of the Effect of Humidity on Recovery

To evaluate the effects of relative humidity on sample collection and recovery, the treated sorbent tubes were placed on an air sampling manifold (Miller-Nelson Flow Temperature Humidity Control System, Model HCS-401) and the flow rate of the manifold was adjusted to 50 cc/min. Each tube was spiked with a solution containing diacetyl, 2,3-pentanedione, 2,3-hexanedione, and 2,3-heptanedione at multiple levels ranging from 0.1 *μ*g to 500 *μ*g (100 *μ*g for 2,3-heptanedione since it was a minor component in all samples). The tubes were allowed to draw laboratory air for two minutes (50 cc/min) to volatilize the analytes of interest before being connected to a Miller-Nelson atmosphere generator. Humidity-controlled air (20%, 50%, and 80%) was sampled for 240 minutes resulting in a total volume of 12 L. The tubes were then refrigerated overnight. To determine whether breakthrough or migration had occurred during sampling, the sorbent from the individual sections of the tubes was removed and placed into individual 4 mL amber colored desorption vials required to prevent UV degradation of samples.

#### 2.3.6. Capacity Studies

The initial collection capacity study was conducted to evaluate the effects of relative humidity on recovery. The custom-made* o*-PDA-treated silica gel tubes were placed on the air sampling manifold and the flow rate of the manifold was adjusted to 50 cc/min. Each tube was then spiked with a mixture containing diacetyl, 2,3-pentanedione, and 2,3-hexanedione at concentrations of 1 *μ*g and 500 *μ*g. Spikes were made on the glass wool preceding the sorbent and humid air was pulled through the tubes.

The tubes were allowed to draw laboratory air for two minutes to volatilize the analytes before being connected to an atmosphere generator to produce the humidity-controlled air (20% and 80%). Humidity-controlled air was sampled for total volumes ranging from 3 L to 24 L (60 to 480 minutes). Sample preparation and analyses were conducted under the parameters previously described.

In an effort to evaluate the effect of an increased sampling rate (200 cc/min) and maximize sampling volumes collected, a more in-depth capacity study was conducted. In this study, 2,3-heptanedione was added as an analyte due to its continued presence as a minor component in alpha-dicarbonyl based flavoring compounds. The custom-made* o*-PDA-treated silica gel tubes were placed on the air sampling manifold and the flow rate of the manifold was adjusted to 200 cc/min. Each tube was then spiked on glass wool at the front section with a solution of diacetyl, 2,3-pentanedione, 2,3-hexanedione, and 2,3-heptanedione at 2 levels (*N* = 3): 0.5 *μ*g and 100 *μ*g. The tubes were allowed to draw laboratory air for two minutes to volatilize the analytes before being connected to an atmosphere generator to produce the humidity-controlled air (20%, 50%, and 80%).

Humidity-controlled air was sampled for total volumes ranging from 96 L to 288 L (480 to 1440 minutes). Tubes were collected from each volume sampled and placed in refrigerated storage overnight. Sample preparation and analyses were conducted under the parameters previously described.

#### 2.3.7. Storage Stability Studies

To evaluate sample stability [13], custom-made* o*-PDA-treated silica gel tubes were spiked with 0.6 *μ*g each of diacetyl, 2,3-pentanedione, 2,3-hexanedione, and 2,3-heptanedione as shown in Table 2. Six sorbent tubes were analyzed after 1, 7, 14, and 30 days. Separate sets of samples were analyzed after storage under ambient and refrigerated storage conditions. Sample preparation and analyses were conducted under the parameters previously described.

## 3. Results

### 3.1. LOD/LOQ Determination

As previously described, eight standards (in duplicate) were analyzed using GC-NPD: diacetyl (2.65 to 662.5 ng/mL), 2,3-pentanedione (10 to 100.7 ng/mL), and 2,3-hexanedione (5 to 201.6 ng/mL). The instrumental LOD and LOQ were determined using calibration curves (diacetyl – slope = 904.66, intercept = 15.74, and *R* ^2^ = 0.9216; 2,3-pentanedione – slope = 533.19, intercept = 29.8, and *R* ^2^ = 0.7918; 2,3-hexanedione – slope = 426.30, intercept = 2.5, and *R* ^2^ = 0.9796; and 2,3-heptanedione – slope = 113.28, intercept = 28.95, and *R* ^2^ = 0.9716). Results are listed in Table 3.

### 3.2. Recovery Study (Untreated Silica Gel Sorbent Tubes)

On the basis of the initial recovery results achieved when diacetyl was spiked directly on untreated silica gel tubes and desorbed in a solution of 1 mg/mL of* o*-PDA, a full scale recovery study was evaluated. Desorption efficiency recoveries for 2,3-dimethylquinoxaline (diacetyl derivative) ranged from 56.3% (0.0955 *μ*g) to 104.3% (478 *μ*g) with an average Relative Standard Deviation (RSD) of 0.039 are listed in Table 4.

### 3.3. Recovery Study (*o*-PDA-Treated Silica Gel Sorbent Tubes)

The next phase in the method development process for the derivatization of diacetyl, 2,3-pentanedione, and 2,3-hexanedione was to determine the feasibility of collecting and derivatizing the analytes “on-tube” using* o*-PDA-coated silica gel sorbent tubes. The initial glass wool plugs were spiked with diacetyl. Ambient air, generated by a Miller-Nelson atmospheric generator, was drawn through the tubes at 0.05 L/min. Desorption efficiencies for the analytes' derivatization with* o*-PDA are depicted in Table 5.

### 3.4. Low-Level Recovery Studies

As noted earlier in the Methods, after the successful recovery study at levels above 1 *μ*g, a low-level recovery study was initiated. The recoveries for diacetyl ranged from 87.2% to 100.7% with an average RSD of 0.073; for 2,3-pentanedione ranged from 94.8% to 120.1% with an average RSD of 0.068; for 2,3-hexanedione ranged from 105.1% to 117.3% with an average RSD of 0.058; and for 2,3-heptanedione ranged from 83.4% to 90.6% with an average RSD of 0.071. Results are listed in Table 6.

### 3.5. Studies of the Effect of Humidity on Recovery

Due to the negative effects that humidity has on diacetyl recovery discovered during some of the more recent field sampling surveys conducted at food and flavoring sites using NIOSH method 2557 [5], the next progression in our method development effort was to evaluate the effect on sample collection of various levels of humidity when using the* o*-PDA-coated silica gel sorbent tubes. Spiking levels ranged from approximately 0.1 *μ*g to 500 *μ*g. After sample collection for a period of 240 minutes and a total volume of 12 L, recoveries were determined for each derivatized analyte collected at relative humidities of 20%, 50%, and 80% (actual measured humidity). The results are listed in Table 7.

### 3.6. Capacity Studies

In the initial collection capacity study, diacetyl, 2,3-pentanedione, and 2,3-hexanedione were sampled on the* o*-PDA-coated silica gel tubes at two levels (1 *μ*g and 500 *μ*g) for total air collection capacities ranging from 3 L (60 min) to 24 L (480 min). Sampling was conducted at relative humidities of 20% and 80% and the results are depicted in Tables 8 and 9.

In an effort to evaluate the effect of an increased sampling rate (200 cc/min) and maximize sampling volumes collected, a more in-depth capacity study was conducted. In this study, 2,3-heptanedione was added as an analyte due to its continued presence as a minor component (contaminant) in alpha-dicarbonyl based flavoring compounds. A more detailed depiction of the recovery data for each analyte is presented in Table 10 (20% RH), Table 11 (50% RH), and Table 12 (80% RH). Mean recovery (%) was calculated based on the average recovery of 3 samples evaluated at each volume sampled.

### 3.7. Storage Stability Recoveries

Evaluation of the ambient and refrigerated storage stability recovery results for diacetyl, 2,3-pentanedione, 2,3-hexanedione, and 2,3-heptanedione indicates that the derivatized analytes were stable for up to 30 days at the 0.6 *μ*g spiking levels. The average storage stability results evaluated for each analyte at 1, 7, 14, and 30 days are reported in Table 13.

## 4. Discussion and Conclusions

A method for alpha-dicarbonyl flavoring compounds has been developed using derivatization with* o*-PDA. This method has several advantages when compared to other methods [3, 6, 7] for alpha-dicarbonyl flavoring compounds, such as improved sensitivity (instrumental LODs of 5–17 ng/sample), use of a single sampling tube amenable to on-tube derivatization of the analytes of interest, longer sampling times, variable sampling rates, and greater sampling capacity (up to 288 L with low-to-moderate humidity). Chromatographic separation of the alpha-dicarbonyl derivatives was good and the overall recovery of the analytes of interest down to the 0.1 *μ*g level was acceptable.

Diacetyl recoveries on untreated silica gel tubes following by desorption in ethanol containing the* o*-PDA derivatizing agent were acceptable at all spiking levels except the lowest (0.096 ng).

Recoveries of diacetyl and 2,3-pentanedione from the silica gel tubes coated with* o*-phenylenediamine were very good while the recoveries for 2,3-hexanedione were approximately 20% lower. Lower recoveries of 2,3-hexanedione and 2,3-heptanedione may be the result of the increasing hydrocarbon nature of these compounds and/or the fact that they possibly require an increased derivatization period. In addition, when larger amounts of the analyte were evaluated, some lower recoveries were found. This may be the result of incomplete derivatization and the need for a greater concentration of the derivatizing reagent on the sorbent media at these higher levels and/or the fact that the higher concentrations evaluated may exceed the sampling capacity of the sorbent tubes. The recoveries for 2,3-hexanedione and 2,3-heptanedione are lower than what is normally considered acceptable [13]. While this method was developed for diacetyl and 2,3-pentanedione measurement to address humidity issues with existing methods, it can be used to determine the presence of larger chain alpha-dicarbonyl compounds that may be present as by-products.

Sample collection for diacetyl was unaffected by humidity ranging from 20% to 80%. For the other flavoring agents tested, high humidity reduced the recovery. While additional research studies are ongoing, it can be reasonably concluded that this method has achieved significant advancements in the sampling and quantitation of alpha-dicarbonyl flavoring compounds. Overall, recoveries were good for diacetyl when sampled in conditions of 80% humidity.

The resulting recovery for 2,3-pentanedione at 95.9 *μ*g (46.2%) is abnormally low when compared to all other results and is most likely an aberration when compared to the results listed in Table 9 for 2,3-pentanedione. Recoveries for both 2,3-hexanedione and 2,3-heptanedione were lower than expected for those samples collected at 80% humidity in the extended air sampling capacity studies. These analytes are present as by-products or contaminants with either diacetyl or 2,3-pentanedione. This method can be used to detect these contaminants where other methods cannot.

In laboratory capacity studies, where diacetyl, 2,3-pentanedione, 2,3-hexanedione, and 2,3-heptanedione were collected at 20% relative humidity and with a sampling rate of 200 cc/min, all three analytes exhibited acceptable recoveries (>80%) with little variation in the mean recoveries when sampling for a total volume of 288 L. At 50% relative humidity, diacetyl, 2,3-pentanedione, 2,3-hexanedione, and 2,3-heptanedione exhibited acceptable recoveries. Results for the samples collected for the 100 *μ*g level showed that 2,3-pentanedione, 2,3-hexanedione, and 2,3-heptanedione had significant decreases in recovery at a collection volume of 216 L. These results would seem to suggest that the maximum sampling volume for these analytes, when collected at a higher sampling rate (200 cc/min), would be between 144 and 216 L.

The average 30-day storage stability recovery results, almost quantitative in nature, are extremely good and acceptable for both the ambient and refrigerated samples of the derivatized diacetyl and 2,3-pentanedione, suggesting that temperature does not have either a positive or a negative effect on the derivatization and storage of the diacetyl samples. Since both the ambient and refrigerated samples had quantitative recoveries (>95%) and RSD values less than 1% [13], there is no difference between the two methods of storage.

Evaluation of both ambient and refrigerated storage stability recovery results for 2,3-hexanedione indicates that the derivatized analyte was stable for up to 30 days at the 0.6 *μ*g spiking levels. Analysis of the results indicates that there is improved storage stability (18% increase in average recovery) when the samples are refrigerated. This is especially true for the samples analyzed after 14 and 30 days. Evaluation of both ambient and refrigerated storage stability recovery results for 2,3-heptanedione indicates that the derivatized analyte was stable for 30 days. Comparison of the averaged recoveries for both the ambient and refrigerated samples of the derivatized 2,3-heptanedione revealed no differences based on storage temperature. Storage stability studies indicated that the compounds of interest, especially diacetyl and 2,3-pentanedione, as their quinoxaline derivatives, are stable at both ambient and refrigerated temperatures for 30 days. Separation of other alpha-dicarbonyls such as 2,3-hexanedione and 2,3-heptanedione can be achieved with this method and provide semiquantitative results. Overall, this method may be another useful tool for the evaluation and monitoring of workers exposed to airborne alpha-dicarbonyl food and flavoring compounds. Additional laboratory and field studies using the method are necessary to obtain full validation and publication in the NIOSH Manual of Analytical Methods (NMAM).

In summary, to date, all results suggest that this method provides the sensitivity needed for nanogram level sampling for alpha-dicarbonyl food and flavoring compounds (diacetyl and 2,3-pentanedione). Additionally, the method allows collection over a wide mass range and at relative humidities ranging from 20% to 80%, with acceptable recoveries achieved up to sampling volumes of 144 L and 288 L for 2,3-pentanedione and diacetyl, respectively. The sampling and analytical methodology has been unaffected by breakthrough when sampling at high flow rates (200 cc/minute) and high sample collection volumes (144 L), eliminating the need for a second sorbent tube in series with the backup section of the single sorbent tube collecting any sample that breaks through. Additionally, the on-tube derivatization eliminates humidity-related breakthrough of the alpha-dicarbonyl flavoring compounds by forming the stable quinoxaline derivatives.

## Figures and Tables

**Table 1:** Preparation of analyte stock solutions and spiking volumes for recovery studies.

Analyte	Spiking level (*μ*g)	Sample volume spiked (*μ*L)	Amount of analyte (*μ*g)
Diacetyl	0.1	0.5	0.0955
	10	50	9.55
	100	10	95.5
	250	25	239
	500	50	478

2,3-Pentanedione	0.1	0.5	0.0959
	10	50	9.59
	100	10	95.9
	250	25	240
	500	50	480

2,3-Hexanedione	0.1	0.5	0.117
	10	50	11.7
	100	10	117
	250	25	292
	500	50	584

**Table 2:** Standard stock spiking solution preparation and spiking volumes for stability studies.

Analyte	Amount neat analyte spike (*μ*L)	Final volume (mL)	Final concentration (*μ*g/mL)	Volume spiked (*μ*L)	Amount spiked (*μ*g)
Diacetyl	1	10	98.5	6	0.591
2,3-Pentanedione	1	10	95.9	6	0.575
2,3-Hexanedione (90%)	1	10	84.0	6	0.504
2,3-Heptanedione	1	10	92.0	6	0.552

**Table 3:** Limit of detection (LOD) and Limit of Quantitation (LOQ) for alpha-dicarbonyl compounds.

Analyte	LOD^14^	LOQ^14^
Diacetyl	7 ng/mL	23 ng/mL
2,3-Pentanedione	17 ng/mL	58 ng/mL
2,3-Hexanedione	5 ng/mL	15 ng/mL

**Table 4:** Diacetyl recovery after extraction of spiked sorbent with *o*-PDA solution.

Spike level (*μ*g)	Average recovery (%)	RSD
0.096	56.3	0.094
9.55	96.4	0.019
95.5	93.0	0.032
239.0	80.1	0.017
478.0	104.3	0.033

**Table 5:** Recovery results for the on-tube derivatization of diacetyl, 2,3-pentanedione, and 2,3-hexanedione.

Analyte	Spike level (*μ*g)	Average recovery (%)	RSD
Diacetyl	0.096	87.0	0.106
	9.55	99.6	0.037
	95.5	91.4	0.099
	239.0	104.0	0.080
	478.0	103.0	0.077

2,3-Pentanedione	0.96^*∗*^	—^*∗*^	—^*∗*^
	9.59	106.5	0.045
	95.9	96.1	0.043
	240.0	92.3	0.064
	480.0	87.3	0.041

2,3-Hexanedione	0.117	69.7	0.212
	11.7	73.5	0.032
	117.0	69.6	0.032
	292.0	66.8	0.040
	584.0	62.5	0.028

^*∗*^Data unavailable due to sample loss during analytical preparation.

**Table 6:** Low level recovery study for diacetyl, 2,3-pentanedione, 2,3-hexanedione, and 2,3-heptanedione.

Analyte	Spike level (*μ*g)	Recovery (%)	RSD
Diacetyl	0.099	87.2	0.139
	0.591	96.7	0.046
	0.985	100.7	0.036

2,3-Pentanedione	0.096	120.1	0.112
	0.575	94.8	0.051
	0.959	108.8	0.038

2,3-Hexanedione	0.084	105.1	0.043
	0.504	117.3	0.056
	0.840	105.1	0.043

2,3-Heptanedione	0.092	83.4	0.072
	0.552	90.5	0.071
	0.920	90.6	0.065

**Table 7:** Effects of varying humidity levels on analyte recovery.

Analyte	Level (*μ*g)	Relative humidity (%)	Mean recovery	RSD
Diacetyl	0.118	16.7	86.4	0.130
	0.118	58.0	101.0	0.146
	0.118	80.1	72.5	0.123
	0.640	22.6	97.7	0.040
	0.640	58.0	111.0	0.098
	0.640	80.4	79.3	0.076
	0.938	20.7	92.4	0.032
	0.938	58.0	115.0	0.121
	0.938	78.5	97.8	0.110
	9.85	17.9	107.6	0.035
	9.85	52.0	100.1	0.076
	9.85	79.6	99.6	0.028
	98.5	17.9	111.0	0.019
	98.5	52.0	100.1	0.076
	98.5	49.6	99.6	0.028
	246.25	17.9	105.0	0.074
	246.25	51.1	101.2	0.087
	246.25	80.6	88.9	0.042
	492.5	17.9	102.7	0.049
	492.5	50.0	108.9	0.037
	492.5	81.0	99.5	0.022

2,3-Pentanedione	0.115	16.7	63.9	0.133
	0.115	58.0	98.7	0.112
	0.115	80.1	73.9	0.123
	0.622	22.6	88.4	0.031
	0.622	58.0	85.6	0.080
	0.622	80.4	76.7	0.074
	0.909	20.7	84.9	0.026
	0.909	58.0	102.0	0.142
	0.909	78.5	98.8	0.137
	9.59	21.1	94.8	0.072
	9.59	51.3	85.8	0.071
	9.59	80.8	64.1	0.107
	95.9	21.1	102.7	0.085
	95.9	51.4	100.0	0.102
	95.9	79.7	46.2	0.060
	239.75	21.1	104.9	0.051
	239.75	50.7	99.1	0.119
	239.75	80.6	73.9	0.110
	479.5	21.1	90.2	0.084
	479.5	50.4	87.1	0.125
	479.5	79.9	80.3	0.112

2,3-Hexanedione	0.101	16.7	120.0	0.195
	0.101	58.0	114.3	0.085
	0.101	80.1	49.9	0.062
	0.546	22.6	108.0	0.038
	0.546	58.0	109.0	0.060
	0.546	80.4	82.4	0.064
	0.799	20.7	103.8	0.034
	0.799	58.0	124.0	0.206
	0.799	78.5	90.8	0.107
	11.68	21.1	67.8	0.055
	11.68	51.3	60.4	0.056
	11.68	80.8	45.4	0.078
	116.75	21.1	66.4	0.051
	116.75	51.4	68.6	0.060
	116.75	79.7	31.1	0.039
	291.88	21.1	66.8	0.028
	291.88	50.7	72.1	0.107
	291.88	80.6	40.4	0.080
	583.75	21.1	59.6	0.057
	583.75	50.4	52.5	0.079
	583.75	79.9	53.7	0.088

2,3-Heptanedione	0.110	16.7	90.3	0.180
	0.110	58.0	69.8	0.142
	0.110	80.1	75.9	0.062
	0.598	22.6	81.0	0.034
	0.598	58.0	88.7	0.066
	0.598	80.4	78.4	0.061
	0.874	20.7	75.3	0.067
	0.874	58.0	108.0	0.195
	0.874	78.5	107.0	0.111
	9.20	21.5	84.9	0.099
	9.20	47.3	84.5	0.053
	9.20	80.3	81.9	0.077
	92.0	21.5	103.1	0.044
	92.0	52.7	92.3	0.030
	92.0	83.8	90.2	0.031

**Table 8:** Determination of air sampling capacity of *o*-PDA-treated silica gel tubes at 20% relative humidity at 2 concentration levels.

Analyte	Level (*μ*g)	Sampling volume (L)	Mean recovery (%)	RSD
Diacetyl	0.985	3	85.5	0.016
	0.985	6	99.9	0.059
	0.985	12	91.1	0.058
	0.985	18	84.0	0.020
	0.985	24	102.9	0.131
	490.0	3	91.1	0.038
	490.0	6	104.6	0.052
	490.0	12	99.0	0.014
	490.0	18	91.8	0.019
	490.0	24	100.4	0.058

2,3-Pentanedione	0.959	3	105.2	0.056
	0.959	6	103.3	0.085
	0.959	12	105.8	0.053
	0.959	18	85.8	0.016
	0.959	24	106.7	0.098
	480.0	3	96.3	0.038
	480.0	6	97.0	0.045
	480.0	12	110.6	0.028
	480.0	18	116.6	0.077
	480.0	24	102.3	0.039

2,3-Hexanedione	0.841	3	72.7	0.087
	0.841	6	91.8	0.077
	0.841	12	110.8	0.109
	0.841	18	85.0	0.016
	0.841	24	92.6	0.114
	420.0	3	89.6	0.036
	420.0	6	86.4	0.089
	420.0	12	90.8	0.059
	420.0	18	110.0	0.032
	420.0	24	108.5	0.103

**Table 9:** Determination of air sampling capacity of *o*-PDA-treated silica gel tubes at 80% relative humidity at 2 concentration levels.

Analyte	Level (*μ*g)	Sampling volume (L)	Mean recovery (%)	RSD
Diacetyl	0.985	3	78.1	0.016
	0.985	6	82.1	0.058
	0.985	12	95.2	0.097
	0.985	18	82.4	0.034
	0.985	24	103.6	0.028
	490.0	3	97.4	0.047
	490.0	6	88.5	0.074
	490.0	12	112.7	0.034
	490.0	18	100.6	0.018
	490.0	24	93.7	0.065

2,3-Pentanedione	0.959	3	100.1	0.030
	0.959	6	76.1	0.051
	0.959	12	101.2	0.068
	0.959	18	97.8	0.048
	0.959	24	81.1	0.139
	480.0	3	97.8	0.055
	480.0	6	92.3	0.085
	480.0	12	99.2	0.058
	480.0	18	87.1	0.010
	480.0	24	85.2	0.071

2,3-Hexanedione	0.841	3	100.7	0.018
	0.841	6	69.5	0.077
	0.841	12	98.3	0.056
	0.841	18	101.4	0.027
	0.841	24	80.4	0.141
	420.0	3	95.5	0.049
	420.0	6	92.5	0.086
	420.0	12	87.7	0.053
	420.0	18	94.7	0.048
	420.0	24	87.6	0.173

**Table 10:** Determination of extended air sampling capacity of *o*-PDA-treated silica gel tubes at 20% relative humidity at 2 concentration levels using increased sampling rate (200 cc/min).

Analyte	Level (*μ*g)	Sampling volume (L)	Time (min)	Mean recovery (%)	RSD
Diacetyl	0.493	96	480	99.8	0.035
	0.493	144	720	110.0	0.040
	0.493	216	960	104.0	0.054
	0.493	288	1440	108.0	0.043
	98.5	96	480	98.3	0.010
	98.5	144	720	104.0	0.023
	98.5	216	960	100.0	0.038
	98.5	288	1440	95.5	0.073

2,3-Pentanedione	0.479	96	480	92.1	0.015
	0.479	144	720	83.0	0.046
	0.479	216	960	101.0	0.088
	0.479	288	1440	109.0	0.181
	95.7	96	480	97.8	0.038
	95.7	144	720	99.4	0.021
	95.7	216	960	103.0	0.156
	95.7	288	1440	95.8	0.046

2,3-Hexanedione	0.420	96	480	97.1	0.021
	0.420	144	720	102.0	0.036
	0.420	216	960	109.0	0.016
	0.420	288	1440	106.0	0.061
	84.1	96	480	87.9	0.036
	84.1	144	720	101.0	0.021
	84.1	216	960	99.4	0.032
	84.1	288	1440	84.6	0.047

2,3-Heptanedione	0.460	96	480	104.0	0.027
	0.460	144	720	101.0	0.065
	0.460	216	960	106.0	0.025
	0.460	288	1440	108.0	0.052
	92.0	96	480	93.9	0.082
	92.0	144	720	98.6	0.029
	92.0	216	960	104.0	0.024
	92.0	288	1440	93.5	0.052

**Table 11:** Determination of extended air sampling capacity of *o*-PDA-treated silica gel tubes at 50% relative humidity at 2 concentration levels using increased sampling rate (200 cc/min).

Analyte	Level (*μ*g)	Sampling volume (L)	Time (min)	Mean recovery (%)	RSD
Diacetyl	0.493	96	480	102.0	0.015
	0.493	144	720	92.6	0.024
	0.493	216	960	102.0	0.018
	0.493	288	1440	87.0	0.026
	98.5	96	480	98.8	0.027
	98.5	144	720	106.0	0.016
	98.5	216	960	101.8	0.020
	98.5	288	1440	101.0	0.028

2,3-Pentanedione	0.479	96	480	92.7	0.010
	0.479	144	720	77.8	0.054
	0.479	216	960	98.5	0.158
	0.479	288	1440	88.5	0.009
	95.7	96	480	97.8	0.030
	95.7	144	720	100.0	0.019
	95.7	216	960	110.0	0.024
	95.7	288	1440	97.8	0.026

2,3-Hexanedione	0.420	96	480	93.7	0.024
	0.420	144	720	86.2	0.021
	0.420	216	960	87.9	0.020
	0.420	288	1440	71.4	0.018
	84.1	96	480	88.0	0.023
	84.1	144	720	102.0	0.023
	84.1	216	960	103.0	0.026
	84.1	288	1440	89.3	0.018

2,3-Heptanedione	0.460	96	480	103.0	0.019
	0.460	144	720	89.3	0.026
	0.460	216	960	97.8	0.031
	0.460	288	1440	98.5	0.041
	92.0	96	480	94.1	0.020
	92.0	144	720	99.8	0.017
	92.0	216	960	105.0	0.011
	92.0	288	1440	98.6	0.019

**Table 12:** Determination of extended air sampling capacity of *o*-PDA-treated silica gel tubes at 80% relative humidity at 2 concentration levels using increased sampling rate (200 cc/min).

Analyte	Level (*μ*g)	Sampling volume (L)	Time (min)	Mean recovery (%)	RSD
Diacetyl	0.985	96	480	99.7	0.017
	0.985	144	720	101.0	0.110
	0.985	216	960	93.7	0.055
	0.985	288	1440	84.1	0.114
	98.5	96	480	90.5	0.072
	98.5	144	720	98.8	0.036
	98.5	216	960	93.5	0.057
	98.5	288	1440	89.4	0.037

2,3-Pentanedione	0.957	96	480	72.9	0.081
	0.957	144	720	112.0	0.055
	0.957	216	960	105.0	0.114
	0.957	288	1440	99.5	0.054
	95.7	96	480	86.0	0.055
	95.7	144	720	93.8	0.038
	95.7	216	960	55.2	0.054
	95.7	288	1440	48.8	0.035

2,3-Hexanedione	0.841	96	480	119.0	0.138
	0.841	144	720	122.0	0.062
	0.841	216	960	105.0	0.114
	0.841	288	1440	44.8	0.031
	84.1	96	480	86.2	0.060
	84.1	144	720	93.5	0.039
	84.1	216	960	60.4	0.053
	84.1	288	1440	54.6	0.015

2,3-Heptanedione	0.920	96	480	52.0	0.021
	0.920	144	720	104.0	0.089
	0.920	216	960	76.0	0.154
	0.920	288	1440	57.2	0.055
	92.0	96	480	85.5	0.058
	92.0	144	720	93.7	0.036
	92.0	216	960	57.8	0.050
	92.0	288	1440	53.7	0.018

**Table 13:** Storage stability conducted under ambient and refrigerated storage conditions.

Day	Avg. recovery (ambient, %)	RSD	Avg. recovery (refrigerated, %)	RSD
Diacetyl				
1	96.1	0.068	96.7	0.046
7	93.4	0.048	89.8	0.039
14	98.6	0.017	106.0	0.065
30	103.0	0.039	98.6	0.047

2,3-Pentanedione				
1	98.0	0.049	94.8	0.051
7	102.0	0.045	100.0	0.025
14	105.0	0.091	97.1	0.068
30	108.0	0.065	96.8	0.073

2,3-Hexanedione				
1	109.8	0.115	117.0	0.056
7	93.9	0.041	110.0	0.054
14	87.0	0.068	99.0	0.040
30	81.4	0.026	99.6	0.053

2,3-Heptanedione				
1	89.3	0.109	90.5	0.072
7	90.8	0.055	84.9	0.059
14	92.2	0.053	83.6	0.096
30	92.2	0.021	97.0	0.082

## References

[1] NIOSH (2006). NIOSH health hazard evaluation report. *NIOSH HETA*.

[2] Kreiss K., Gomaa A., Kullman G., Fedan K., Simoes E. J., Enright P. L. (2002). Clinical bronchiolitis obliterans in workers at a microwave-popcorn plant. *The New England Journal of Medicine*.

[3] Pendergrass S. M. (2004). Method development for the determination of diacetyl and acetoin at a microwave popcorn plant. *Environmental Science and Technology*.

[4] Ashley K., McKernan-Taylor L., Burroughs E., Deddens J., Pendergrass S. M., Streicher R. P. (2008). Analytical performance criteria. *Journal of Occupational and Environmental Hygiene*.

[5] Cox-Ganser J., Ganser G., Saito R. (2011). Correcting diacetyl concentrations from air samples collected with NIOSH method 2557. *Journal of Occupational and Environmental Hygiene*.

[6] OSHA (2003). *OSHA Method PV2118: Acetoin and Diacetyl*.

[7] OSHA (2008). *OSHA Method 1013: Acetoin and Diacetyl*.

[8] NIOSH (2011). *Occupational Exposure to Diacetyl and 2,3-Pentanedione*.

[9] OSHA (2008). OSHA Method 1012: Acetoin and Diacetyl. U.S. Department of Labor, Occupational Safety and Health Administration.

[10] Fujioka K., Shibamoto T. (2006). Determination of toxic carbonyl compounds in cigarette smoke. *Environmental Toxicology*.

[11] Barros A., Rodriques J. A., Almeida P. J., Oliva Teles M. T. (1999). Determination of glyoxal, methylglyoxal, and diacetyl in selected beer and wine, by HPLC with UV spectrophotometric detection, after derivatization with o-phenylenediamine. *Journal of Liquid Chromatography & Related Technologies*.

[12] Rodrigues J. A., Barros A. A., Rodrigues P. G. (1999). Differential pulse polarographic determination of *α*-dicarbonyl compounds in foodstuffs after derivatization with *o*-phenylenediamine. *Journal of Agricultural and Food Chemistry*.

[13] Kennedy E. R., Fishback T. J., Song R., Eller P. M., Shulman S. A. (1995). *Guidelines for Air Sampling and Analytical Method Development and Evaluation*.

